# The Indirect Mineral Carbonation of Electric Arc Furnace Slag Under Microwave Irradiation

**DOI:** 10.1038/s41598-019-44162-x

**Published:** 2019-05-22

**Authors:** Zhibo Tong, Guojun Ma, Dan Zhou, Gang Yang, Cheng Peng

**Affiliations:** 1 The Key Laboratory of Extraordinary Bond Engineering and Advanced Materials Technology, Yangtze Normal University, 408100 Chongqing, China; 2 Metallurgical Secondary Resources Engineering Technology Research Center, Wuhan University of Science and Technology, 430081 Wuhan, China

**Keywords:** Environmental sciences, Natural hazards

## Abstract

The indirect mineral carbonation of industrial residues is one of the potential technologies for CO_2_ sequestration. In this paper, the leaching and carbonation of electric arc furnace (EAF) slag under microwave irradiation was investigated. The experimental results show that the main reactive calcium-containing phase in the EAF slag carbonation process is calcium silicate, and the final leaching ratio of larger particles is lower than that of smaller particles due to the silica layer produced on the surface of the calcium silicate. The Drozdov equation with a self-impeding coefficient can describe EAF slag leaching kinetics under microwave irradiation. The explosive homogeneous nucleation phenomenon under microwave irradiation contributes to the thinning and narrowing of crystals. Microwave irradiation can inhibit the crystaltype transformation of vaterite.

## Introduction

With the modernization and industrialization of countries over the past centuries, large amounts of CO_2_ emissions and industrial solid waste have caused severe pollution^1^. Although the use of fossil fuels as the primary sourceof the world’s energy results in high CO_2_ emissions, their usage is inevitable in the foreseeable future due to the costs of other energy sources, societal pressures and the established infrastructure^1–5^. To effectively reduce artificial CO_2_ emissions, carbon capture and storage (CCS) is widely used. Mineral carbonation, a potentially important technology in CCS, has many advantages^6–8^. For example, the process energy consumed by mineral carbonation is very low, and product of the process is stable in nature. Since the weathering reaction of silicate and CO_2_ requires a large geological time scale, this reaction is not suitable for CO_2_ sequestration on engineering time scales. Therefore, many solutions have been developed to improve the reaction rate and reduce the process cost, such as adopting a wide variety of materials, leaching agent and methods.

In the past three decades, natural minerals, such as orsterite, serpentine and wollastonite^9–12^, have been used as mineral carbonation feedstocks. With the development of research, some industrial wastes, such as metallurgy slag, fly ashes, and building and mining waste, have been applied as feedstocks^13–19^ because these industrial wastes are more active and do not need to be exploited. Moreover, industrial wastes are usually close to CO_2_ emission sites, so the transportation cost can be greatly decreased.

As the dry reaction is usually much slower than the wet reaction, the study of mineral carbonation focuses on wet reaction^20,21^. In the case of aqueous mineral carbonation, leaching agents have attracted more attention because lower pH values are helpful for the leaching of alkaline earth elements but harmful for CO_2_ fixation. Strong acids, such as hydrochloric acid, were first selected as leaching agents^9,10^, and then weak acids were gradually used as leaching agents, based on thermodynamic considerations^22^; however, impurity ions are the main drawback, and carbonate precipitation is difficult to generate without a pH increasing agent in some cases^18^. Subsequently, ammonium salts^23^ have been studied as leaching agents due to their lower acidity. Afterthe leaching reaction, the system pH scale of ammonium chloride solution ranges from 8.0~10.5, where only calcium ions exist, so pure calcium carbonate can be prepared without purification. Additionally, ammonium salts used as leaching agents can be recycled at the carbonation stage, so the process effectively reduces the cost and has attracted much attention. Nevertheless, the leaching ratio is far from satisfactory. However, many methods have also been proposed to improve the leaching ratio. Park and Fan^24^ found that the fluidization of serpentine slurry with 2 mm glass beads was most effective in refreshing the surface of the serpentine particles during the dissolution process. Finely ground and heat-activated serpentine derived from mining residues has also been studied for CO_2_ capture and sequestration^25^. Additionally, some unconventional methods, such as ultrasonic and microwave treatment, have been shown to be effective for leaching^26–28^. Our previous study showed that microwave irradiation can improve the calcium leaching ratio by approximately 10%^28^; however, the leaching mechanism requires further research.

To date, there has been little attention on the influence of microwaves on the preparation of calcium carbonate by bubbling carbonation in leaching systems, but the authors think that a study on the production of calcium carbonate under microwave irradiation is of interest. Microwave irradiation has already been used in carbonation reactions in other systems. For example, Rodríguez and Gómez^10^ believed that microwave irradiation provides a driving force for homogeneous nucleation. Another example is carbonation crystallization under microwave irradiation at 80 °C and 90 °C, which was studied by Rizzuti and Leonelli^29^, and the results indicated that microwave irradiation has a substantial influence on the crystal form of calcium carbonate. Furthermore, microwave irradiation not only is helpful for the crystallization reaction but also has the effect of thinning and narrowing crystal particles.

In this study, electric arc furnace (EAF) slags were used as the experimental feedstock, and ammonium chloride solution was used as the leaching agent during the mineral carbonation process. Many researchers have reported the effect of different methods on mineral carbonation; all of the studies focused on how to improve the leaching and carbonation rate and ratio. Furthermore, to further study the leaching mechanism under microwave irradiation in this study, we also investigated the difference in carbonation processes under microwave irradiation and under a traditional approach in the NH_4_Cl-NH_3_-H_2_O system. Moreover, to broaden the application of microwave irradiation in mineral carbonation, the leaching kinetics and the characteristics of calcium carbonate produced under microwave irradiation were discussed.

## Experimental Procedure

### Sample and preparation

Electric arc furnace (EAF) steelmaking slags that had undergone magnetic separation were obtained from a local steelmaking plant, and the major chemical composition of the slag is shown in Table 1. The slag samples were comminuted in a vibratory disk mill and then dried in an oven, after which the slags were sieved into four size fractions. The calcium oxide contents of the four size fractions were analyzed with inductively coupled plasma emission spectroscopy (IRIS Advantage ER/S, Thermo Jarrell Ash, US), and the results (in Table 2) show that the CaO mass percentages (wt%) of the four size fractions are similar.

**Table 1:** Composition of electric arc furnace steelmaking slag.

Slag component	CaO	Fe_2_O_3_	SiO_2_	MgO	Al_2_O_3_	Mn	P	S	C	IF
Mass percentage (wt%)	39.04	28.36	12.54	8.79	4.18	0.9	0.45	0.29	1.49	5.54

**Table 2:** Calcium oxide contents of the four size fractions.

Size Fraction (μm)	54~74	74~97	97~150	150~340
Mass percentage (wt%) of CaO	37.84	38.11	38.51	39.6

### Experimental procedure

The study was divided into two stages: (a) leaching experiments of the four size fractions under microwave irradiation and (b) carbonation experiments in a water bath and under microwave irradiation. The MCR-3 microwave chemical reactor can provide microwave irradiation with stable temperature or power. In the leaching experiment, 17 g EAF slag was leached by 340 mL ammonium chloride solution (2 mol/L) at 60 °C under microwave irradiation. During the experiment, approximately 2 ml of solution was removed by syringe at the designed time and filtered immediately. The calcium content in the filtrates was titrated with EDTA. The filtered residues were triply washed with reverse osmosis water and dried for study by using a field emission scanning electron microscope (Nova 400 Nano SEM, FEI, Hillsboro, OR, USA)-energy-dispersive spectrometer (EDS) (INCAIE 350 Penta FET X-3 EDS, Oxford, UK). As-received EAF slag samples were also analyzed with SEM-EDS.

The carbonation experiments were performed in a water bath with different temperatures and under microwave irradiation with different powders. High-purity CO_2_ gas was bubbled into a mixed solution (2.4 mol/l NH_4_Cl-0.8 mol/l CaCl_2_-1.6 mol/l NH_3_·H_2_O, 400 ml). A 2 ml a liquot of the reaction slurry was transferred to the filter by syringe at the designed sampling time. The calcium ions in the filtrate were titrated with EDTA, the filtered residues were triply washed with distilled water and rinsed with 20~30 ml ethanol, and the washed solutions were then processed with ultrasonic shaking for ten minutes. Then, the calcium carbonate particle sizes in the processed solutions were measured immediately with a Malvern Mastersizer 2000, and the mineralogical phases of the calcium carbonate were determined by an X’Pert PRO MPD X-ray diffractometer with Cu Kα radiation. The produced calcium carbonate was studied using a field emission scanning electron microscope (Nova 400 Nano SEM, FEI, Hillsboro, OR, USA)-energy-dispersive spectrometer (EDS) (INCAIE 350 PentaFET X-3 EDS, Oxford, UK).

## Results and Discussion

### The mechanism of the leaching reaction

**Figure 1:**
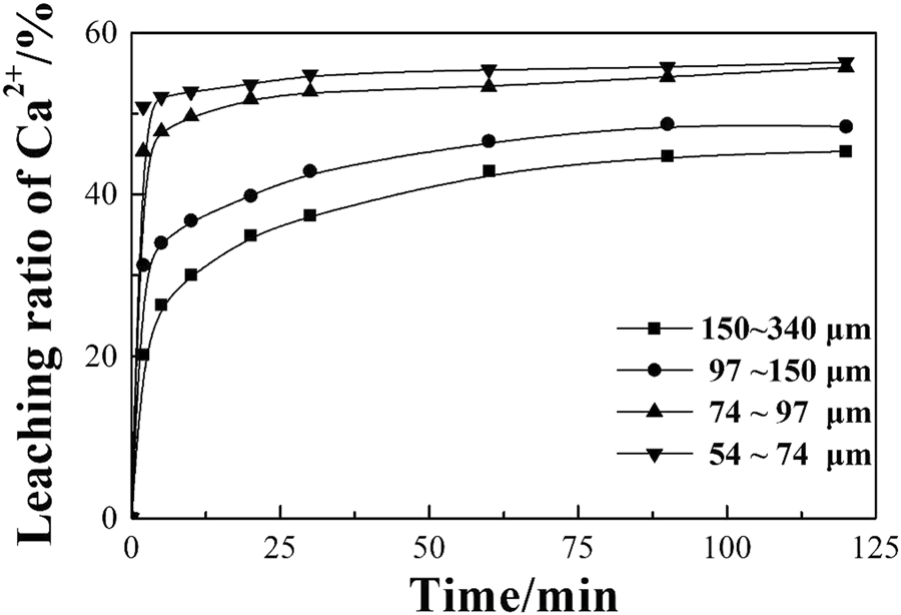
Leaching process of particles with different sizes.

**Figure 2:**
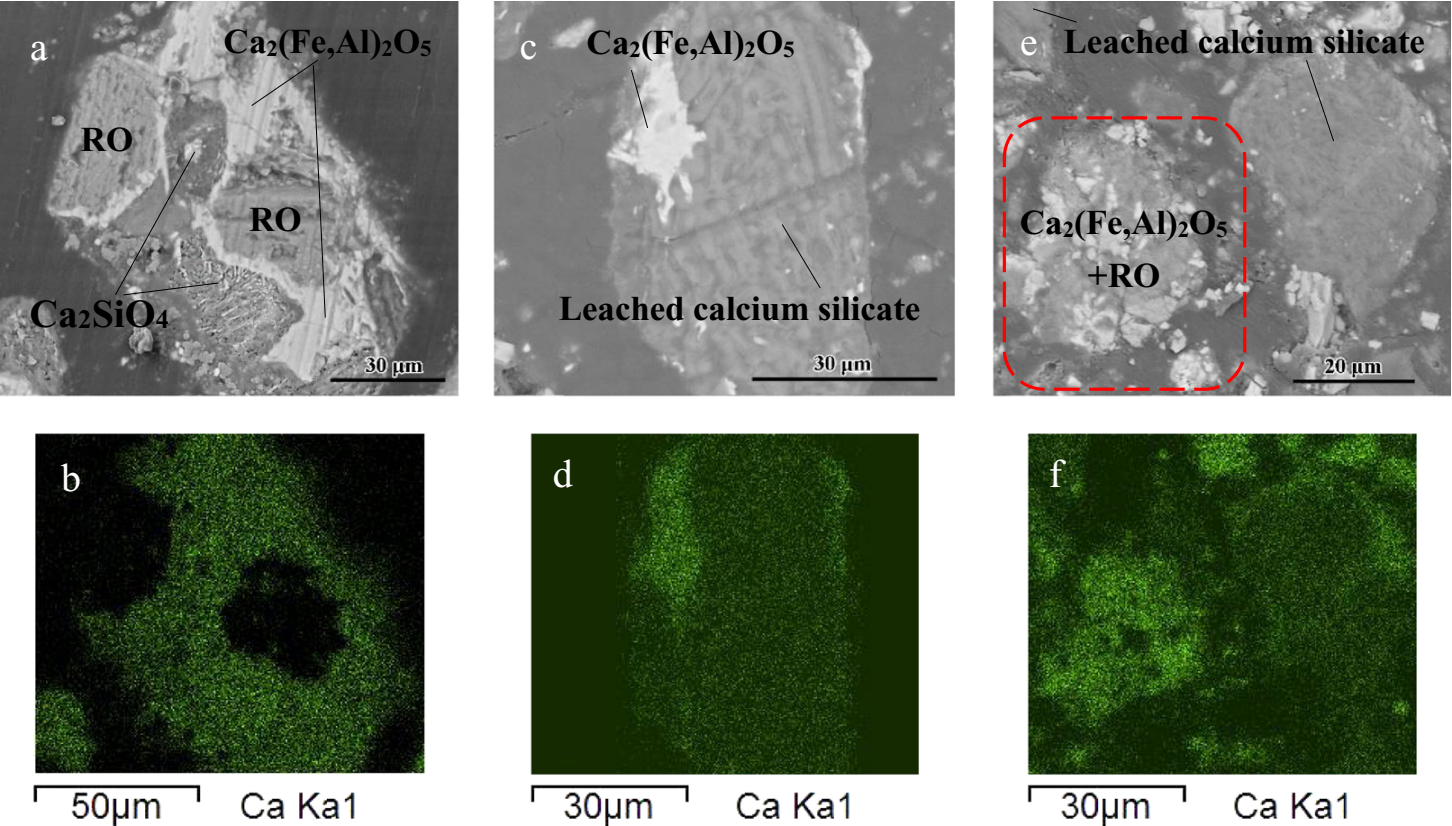
Metallographic microstructure of typical EAF slag and leached EAF slag.

From the leaching ratio curves of the four size fractions in Fig. 1, the initial leaching ratio of calcium was much lower, and the final leaching ratio was also lower for larger particles. Although the leaching ratio tends to reach a balance as the reaction time increases for each particle size, small particles clearly tend to reach a balance more quickly. As the phases of EAF slag are complex and their leaching behaviors are different, comparing the changes in the main phases before and after the leaching reaction will be helpful in understanding the leaching mechanism. Preliminary SEM and XRD analysis indicated that the main phases in EAF slag are calcium silicate, calcium ferroaluminates and the RO phase, where the RO phase is a solid solution mainly containing iron and magnesium without calcium^28^. Figure 2a,b shows typical metallographic SEM images of three-phase symbiosis. Figure 2b shows that calcium mainly exists in calcium silicate and calcium ferroaluminates. Typical metallographic microstructures of leached residues at 54~74 μm are shown in Fig. 2c–f. It can be seen that calcium is leached from silicate instead of ferroaluminates from leached residues at 2 min (Fig. 2c,d), and the calcium ferroaluminates still do not react when the leaching reaction reaches 120 min (Fig. 2e,f), so the main reactive calcium-containing phase in EAF slag is only calcium silicate.

**Figure 3:**
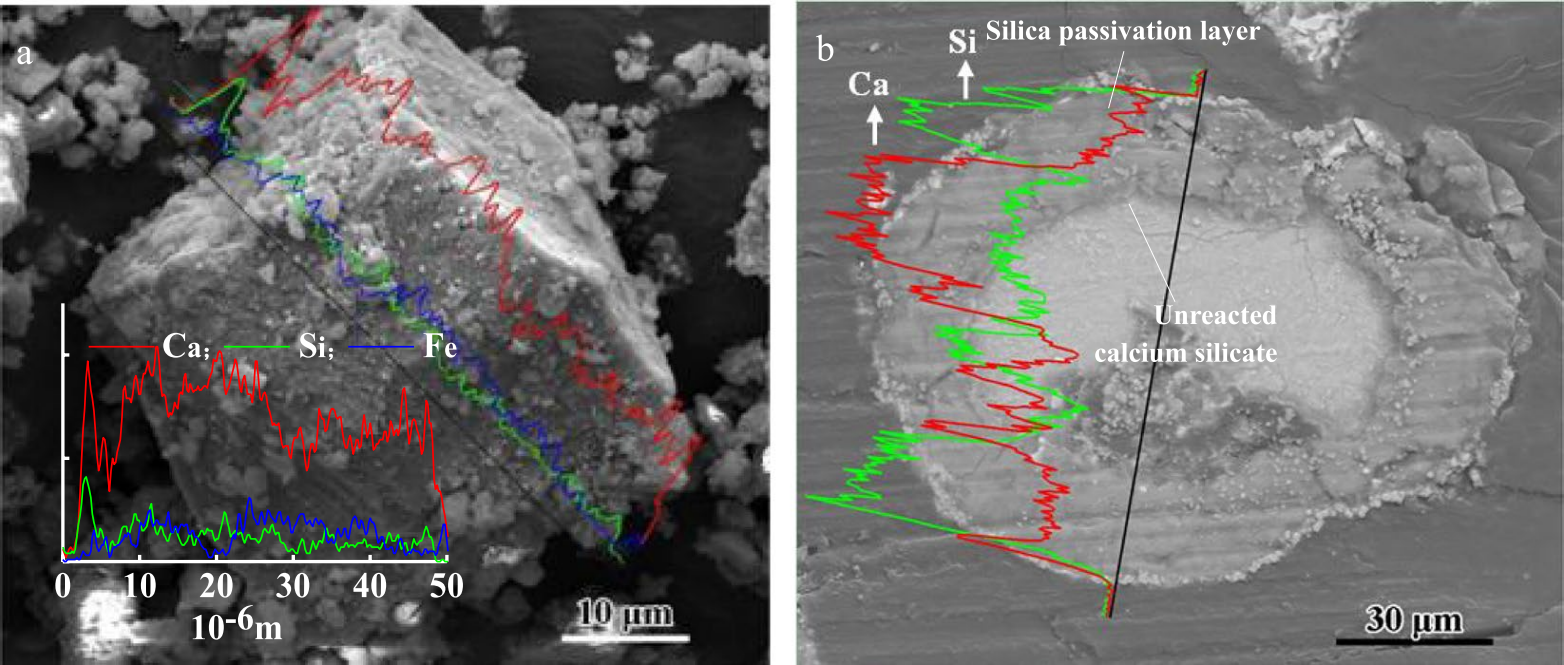
SEM images of calcium silicate in as-received slag and leached slag.

Figure 3a shows the as-received slag, in which the calcium silicate surface is smooth and dense and the calcium content is high. In contrast, in the 2 min leached slag in Fig. 3b, there is a loose silica passivation layer produced on the surface of calcium silicate particles (97~150 μm), and inpractice, the leaching reaction will be prevented as the passivation layer becomes thicker with increased leaching time.

**Figure 4:**
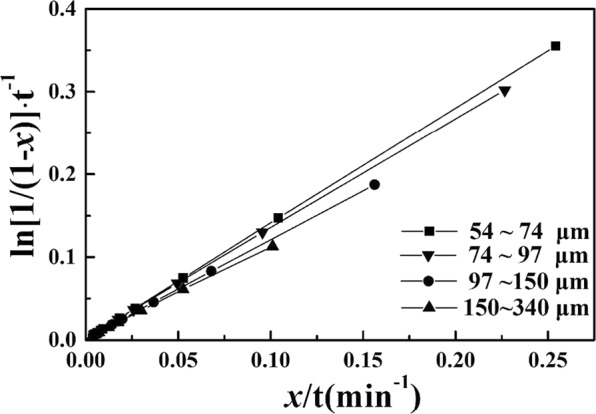
The relationship between ln[1(1 − *x*)]·t^−1^ and xt for different grain sizes.

According to the leaching curve in Fig. 1, the leaching ratio of calcium increases quickly in the beginning and tends to reach a balance later, which clearly indicates a resistance phenomenon throughout the whole process. Putting the data from Fig. 1 into the kinetic model of the Drozdov equation with a self-impeding coefficient as below, the results from Fig. 4 show that ln[1/(1 − *x*)]·t^−1^ and *x*/t have a strong linear correlation with a correlation coefficient of more than 0.999. This result indicates that the Drozdov equation with a self-impeding coefficient can describe EAF slag leaching kinetics under microwave irradiation.

$$\frac{1}{t}\,\mathrm{ln}\,\frac{1}{1-x}-\beta \frac{x}{t}={K}_{m}$$

where *t* is the leaching time, min; *x* is the leaching ratio, %; *β* is the self-impeding coefficient; and *K*_m_ is the reaction ratio constant.

### Carbonation under microwave irradiation

**Figure 5:**
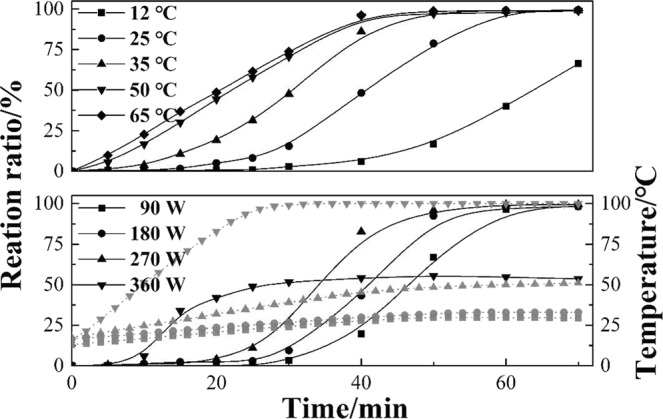
The calcium carbonate reaction ratio and heating curve of the solution with time.

Figure 5 shows the calcium carbonate reaction in a water bath and under microwave irradiation. The reaction ratio was only 66% at 12 °C in the water bath, and the reaction ratio accelerated with increasing temperature. The reaction time was reduced to 50 min due to the rate of diffusion, and the reaction accelerated when the temperature reached 50 °C^30^. However, the reaction curve did not change much as the temperature increased to 65 °C, probably because evaporating ammonia affects the dissolution of CO_2_. Furthermore, microwave irradiation improves the reaction rate; however, the solution boils in 30 min, and the reaction stops when the microwave power reaches 360 W. Interestingly, in contrast to the experiments in a water bath, the reaction ratio under microwave irradiation is very low at the initial stage and then increases rapidly until reaching the end of the reaction. The phenomenon of explosive homogeneous nucleation contributesto the thinning and narrowing of crystals.

**Figure 6:**
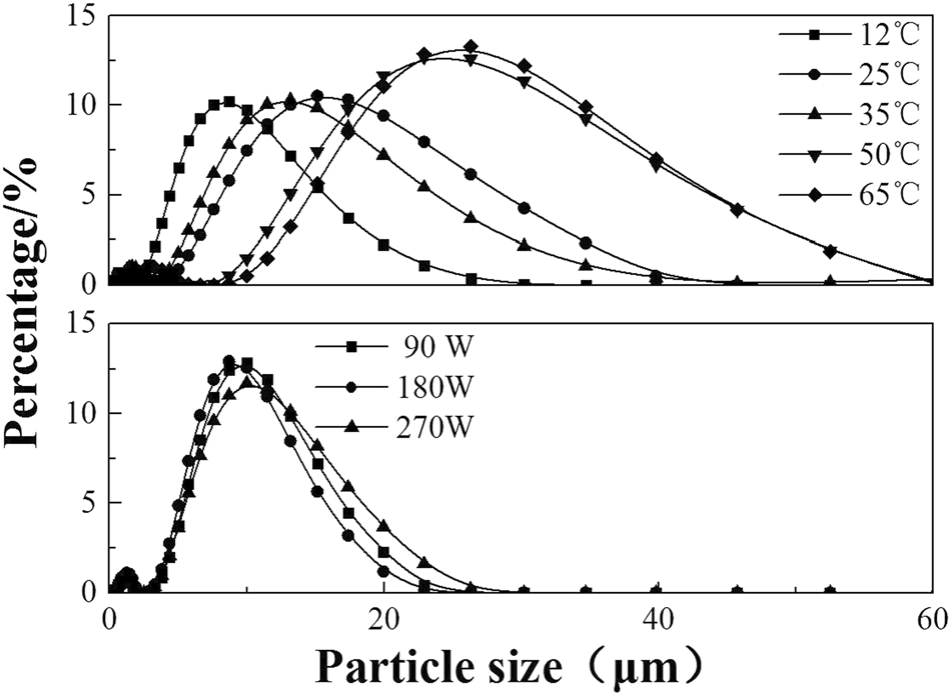
The effect of temperature and microwave power on particle size.

**Figure 7:**
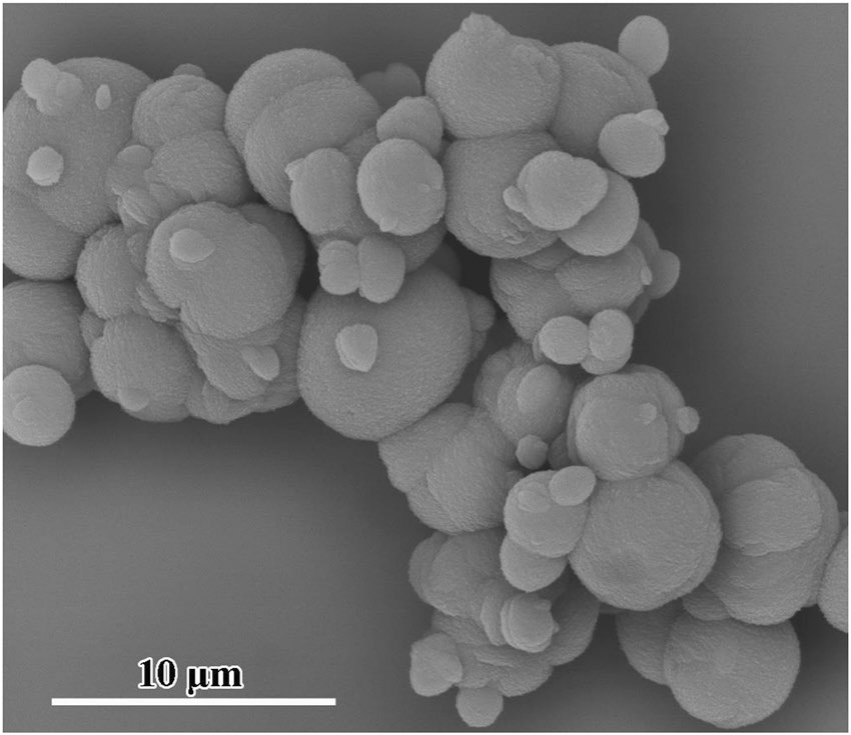
Typical SEM image of calcium carbonate produced by reaction under microwave irradiation.

The particle size of the produced calcium carbonate was analyzed after the carbonation reaction, as shown in Fig. 6. In a water bath, the particle size increases with increasing temperature, and the distribution range becomes broadened. However, the change in particle size is small under microwave irradiation, and the particle size of the produced calcium carbonate is considerably smaller and more narrow than that obtained in a water bath (except at 12 °C), which agrees with the phenomenon of explosive homogeneous nucleation in Fig. 5. In addition, there are two peaks in Fig. 6, which is the same as in a report about the carbonation of calcium hydroxide slurry^31^. The smaller peak is the primary crystals with a particle size of 0.4~2.5 μm, and the larger peak is the agglomeration of many initial crystallites, as indicated by the typical SEM image (Fig. 7).

**Figure 8:**
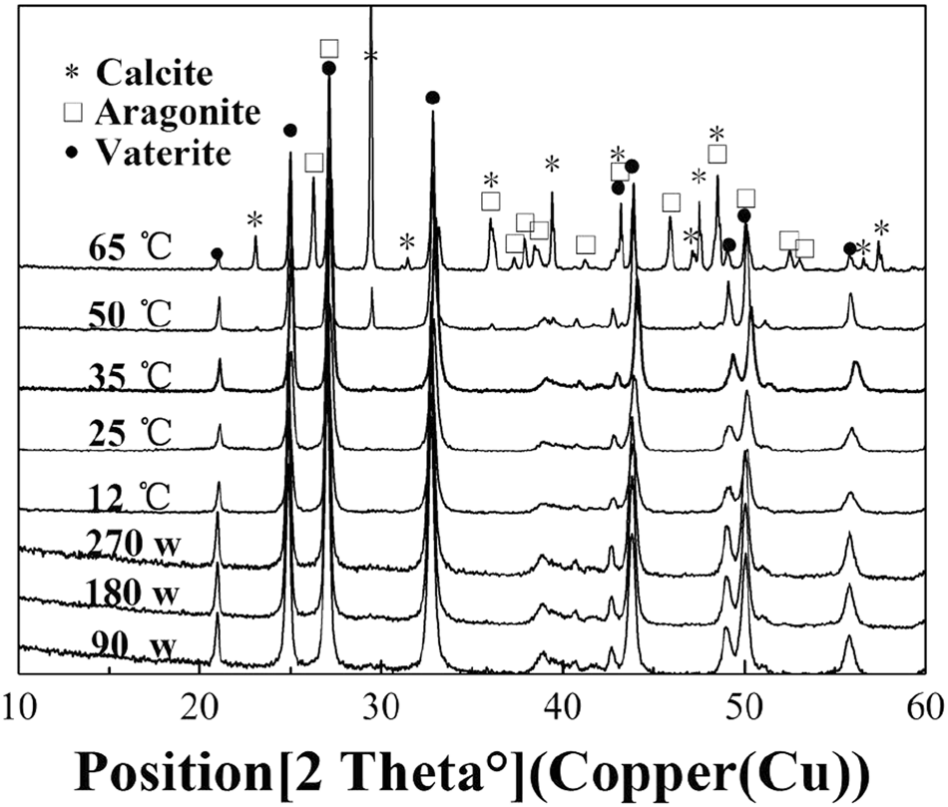
X-ray diffraction patterns of calcium carbonate.

**Figure 9:**
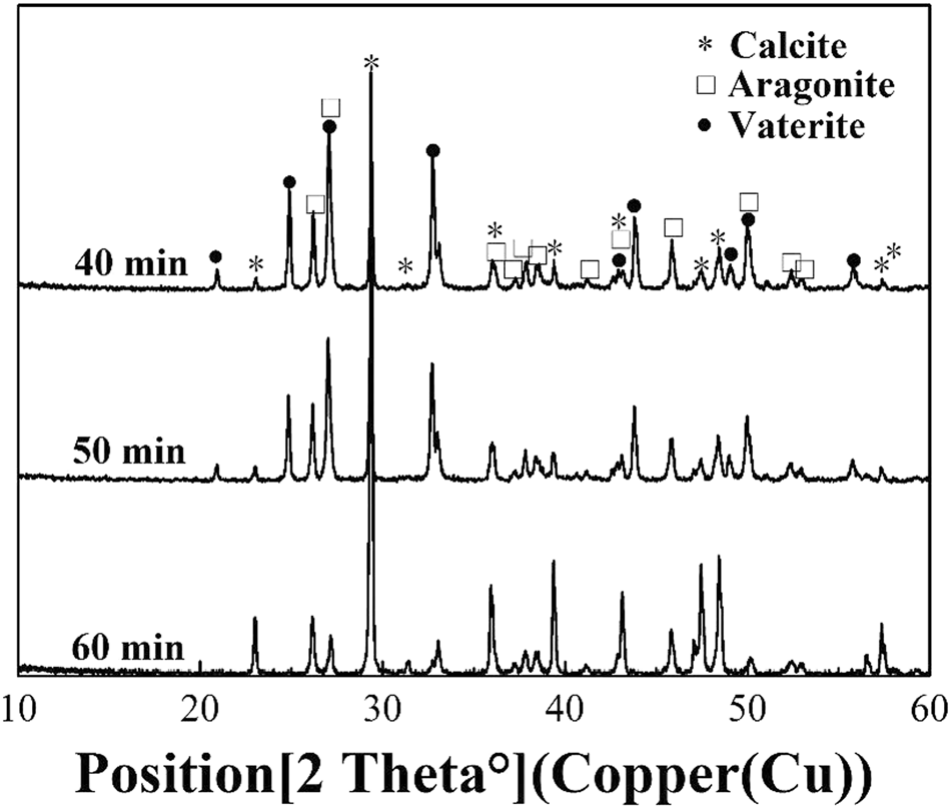
X-ray diffraction patterns of calcium carbonate with different time at 65° C.

As shown in Fig. 8, there was only vaterite at 12, 25 and 35 °C in the water bath, which is consistent with the report that vaterite is produced in ammonium chloride-ammonia^32,33^. However, the calcium carbonate crystals gradually converted into aragonite and calcite as the temperature increased to 50 and 65 °C, which indicates that temperature has a strong influence on the morphology of calcium carbonate and that the temperature should remain beneath a threshold for the preparation of pure calcium carbonate products. This conclusion is similar to the results of Tom Van Gerven’s study^18^, which considered the preparation of high-quality precipitated calcium carbonate using a blast furnace (BF). Moreover, it can be seen from Fig. 9 that vaterite, aragonite and calcite have been produced at temperature of 65° C, and then the produced calcium carbonate gradually turn into calcite crystal with the increase of reaction time. In contrast, all the calcium carbonate crystals prepared under different powers of microwave irradiation were vaterite, although the solution temperature was relatively high (about 50 °C) during half of the reaction time for a microwave power of 270 W (Fig. 5), which is a powerful statement that microwave irradiation can inhibit the crystalty petrans formation of vaterite.

## Conclusions

The main reactive calcium-containing phase is calcium silicate in the EAF slag carbonation process, and the final leaching ratio of larger particles is lower than that of smaller particles due to the silica layer produced on the surface of calcium silicate. The Drozdov equation with a self-impeding coefficient can describe EAF slag leaching kinetics under microwave irradiation. The explosive homogeneous nucleation phenomenon under microwave irradiation contributes to the thinning and narrowing of crystals. Microwave irradiation can inhibit the crystaltype transformation of vaterite.
